# The cholera challenge: How should the world respond?

**DOI:** 10.1016/j.nmni.2022.101077

**Published:** 2022-12-23

**Authors:** Jaffar A. Al-Tawfiq, Hitesh Chopra, Kuldeep Dhama, Ranjit Sah, Patricia Schlagenhauf, Ziad A. Memish

**Affiliations:** Infectious Disease Unit, Specialty Internal Medicine, Johns Hopkins Aramco Healthcare, Dhahran, Saudi Arabia; Division of Infectious Diseases, Indiana University School of Medicine, Indianapolis, IN, USA; Division of Infectious Diseases, Johns Hopkins University, Baltimore, MD, USA; Chitkara College of Pharmacy, Chitkara University, Punjab, India; Division of Pathology, ICAR-Indian Veterinary Research Institute (IVRI), Izatnagar, 243122, Bareilly, Uttar Pradesh, India; Tribhuvan University Teaching Hospital, Institute of Medicine, Kathmandu, Nepal; Dr. Y Patl Medical College, Hospital and Research Center, Dr. D.Y. Patil Vidyapeeth, Pune, Maharashtra, India; WHO Collaborating Centre for Travellers' Health, Institute for Epidemiology, Biostatistics and Prevention, University of Zürich Centre for Travel Medicine, MilMedBiol Competence Centre, University of Zürich, Switzerland; Al-Faisal University, Riyadh, Saudi Arabia; King Saud Medical City, Ministry of Health, Riyadh, Saudi Arabia; Hubert Department of Global Health, Rollins School of Public Health, Emory University, Atlanta, GA, USA

**Fig. 1:**
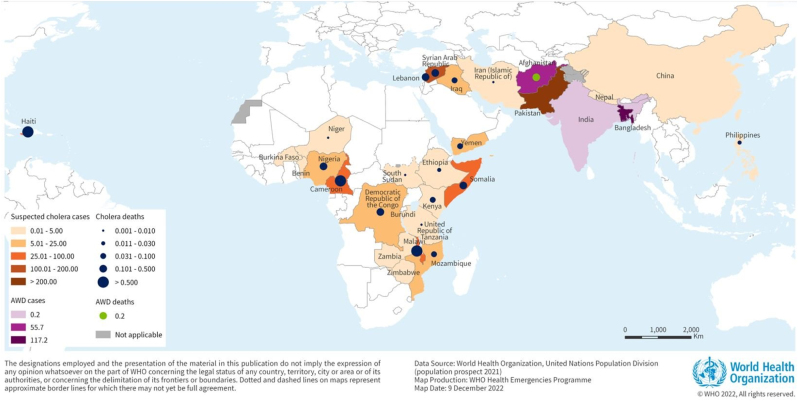
Incidence of cholera cases (including estimated cases of acute watery diarrhea (AWD)iv) per 100,000 population reported to WHO from 1 January to 30 November 2022. Figure is from the World Health Organization: https://www.who.int/emergencies/disease-outbreak-news/item/2022-DON426.

Cholera is an acute diarrheal disease that spreads via contaminated food and water, and can be lethal if not treated timely. It is caused by toxigenic strains of Gram-negative bacterium *Vibrio cholerae*, a category B agent, which is endemic in 47 less developed countries primarily in Africa and South and South-East Asia [1,2]. There were 23 countries in 2021 and 29 countries in 2022 reporting the occurrence of new cholera cases as reported to the World Health Organization (WHO) (Fig. 1) [3]. In addition, 16 of these countries reported prolonged outbreaks as November 30, 2022 [3]. According to a recent WHO report, the drivers for the current Cholera outbreaks include: “widespread floods and drought, humanitarian crises, political instability, and conflict, Multiple ongoing emergencies, sub-optimal/delayed surveillance, and insufficient availability of oral cholera vaccine [3]. In addition, one study showed that environmental isolates of the cholera bacterium showed extensive genomic rearrangements, presence of mobile genetic elements with many breakpoints, relocations, and insertions [4]. It is estimated that there are an annual 1.3-4.0 million Cholera cases with 21,000–143,000 deaths globally [5]. Thus, in 2017 WHO launched a strategy to end global Cholera by 2030 with the aim to decrease the death rates due to this bacterial infection by 90% [6].

Cholera is endemic in Iraq and Syria and its periodic outbreaks have been recorded since 1966 [7] along with recent outbreaks reported from both countries wherein few deaths among confirmed cases were recorded as of September 2022. A recent outbreak of cholera occurred in Lebanon and this is the first since about 30 years with a total of 18 cases reported as of October 21, 2022. In addition, three sewage stations tested positive for cholera in Ain Mraisseh in Beirut, Ghadir station in Mount Lebanon, and Bourj Hammoud in Mount Lebanon [8]. The findings showed that cholera had already spread to Beirut Area and Mount Lebanon, away from the cases [8]. The occurrence of these cases is of concern especially with regard to the possible spread of cholera to neighboring countries. The relation of the outbreak in Lebanon to an ongoing cholera outbreak in Syria reported a few weeks earlier is still unknown [8]. Earlier outbreaks of cholera in mass gatherings had resulted in multiple outbreaks during the Hajj in 1984-86 and 1989 [[9], [10], [11]]. These outbreaks were due to inadequate food hygiene, asymptomatic carriers of bacteria, and mass food preparation [11]. However, the Kingdom of Saudi Arabia had invested in upgrading the water and waste system infrastructure and setup strict regulations restricting the import of food with pilgrims and controlling the food preparation in Hajj premises [12]. A recent study showed no cholera among French pilgrims in 2016-2017 [13]. Due to the fact that cholera continues to cause global outbreaks in many countries, this infection continues to be under continuous surveillance during the Hajj based on proactive surveillance, early detection and implementation of prompt control measures [9]. Outbreaks of cholera have rarely been reported in developed nations. However, cases are typically imported from endemic or epidemic nations to cholera-free countries by travelers [14,15]. World Cup in Qatar 2022 is the most immediate mass gathering events occurring as the Cholera outbreak is ongoing in 26 countries across the globe. For travelers, it is important to follow the proper preventive measures in order to minimize the risk of *Vibrio cholerae* infection and seek prompt medical services when diarrhea is developed [16]. However, it is important to keep in mind the possibility of the occurrence of Cholera and the need for continued vigilance, surveillance and improving sanitary and hygiene practices, follow frequent hand-washing practices, use safe water and adopt appropriate food safety measures. This includes very stringent surveillance at ports of entry and increasing awareness of all health care workers about the disease, in addition to prompt testing ability using culture and PCR, and the ability to quarantine contacts. Treatment options include oral rehydration and antibiotics for checking diarrhea and counteract associated morbidity and mortality.

There are three killed whole-cell oral cholera vaccines. These are Dukoral (Valneva, Stockholm, Sweden), Shanchol (Shantha Biotechnics, Hyderabad, India), and Euvichol (EuBiologics, Seoul, South Korea) [17]. The oral cholera vaccines (OCVs) have >60% effectiveness against cholera disease. There is a significant global shortage of cholera vaccine leading the WHO to recently recommending a single dose vaccination instead of two. In addition, for the control of Cholera outbreaks, the WHO considers vaccination especially for individuals in areas/hotspots where vaccination is most needed to reach high vaccination rates and to prevent further spread of the *V. cholerae* in communities, but as of the current time no recommendation is made to use cholera vaccine for travellers from endemic or countries with ongoing outbreaks of cholera [16]. However, the issue of vaccine is further complicated by the shortage of vaccine supply and the lack of availability of safe drinking water for a large population of the world [18]. This needs to be revisited in the case of travellers from cholera affected countries attending mass gathering events where the risk of igniting outbreaks is significant.

Exploring advanced diagnostics, surveillance and monitoring tools, developing more efficacious vaccines and drugs would facilitate the global roadmap for effectively preventing and controlling cholera outbreaks along with limiting its transmission and spread in hotspots regions via following appropriate vaccination coverage, checking emerging drug resistance, strengthening of adequate sanitation and hygiene, providing safer water supplies, limiting sewage contamination of drinking water, formulating proactive control strategies and preparedness plans to combat cholera outbreaks, would altogether help in achieving the global goal to decrease deaths due to cholera deaths by up to 90% in coming years to come [2,19].
